# Prenatal and postnatal tobacco smoke exposure and respiratory health in Russian children

**DOI:** 10.1186/1465-9921-7-48

**Published:** 2006-03-28

**Authors:** Jouni JK Jaakkola, Anna A Kosheleva, Boris A Katsnelson, Sergey V Kuzmin, Larissa I Privalova, John D Spengler

**Affiliations:** 1 Institute of Occupational and Environmental Medicine, University of Birmingham, Birmingham, UK; 2 Department of Environmental Health, Harvard School of Public Health, Boston MA, USA; 3 The Urals Regional Center for Environmental Epidemiology (URCEE), Ekaterinburg, Russia

## Abstract

**Background:**

Only few studies have assessed the relative impact of prenatal and postnatal exposure to tobacco smoke on the child's later asthma or chronic respiratory symptoms and to our knowledge no studies have elaborated respiratory infections and allergies in this context.

**Objective:**

To assess the effects of prenatal and postnatal exposure to tobacco smoke on respiratory health of Russian school children.

**Methods:**

We studied a population of 5951 children (8 to12 years old) from 9 Russian cities, whose parents answered a questionnaire on their children's respiratory health, home environment, and housing characteristics. The main health outcomes were asthma, allergies, chronic respiratory symptoms, chronic bronchitis, and upper respiratory infections. We used adjusted odds ratios (ORs) from logistic regression analyses as measures of effect.

**Results:**

Prenatal exposure due to maternal smoking had the strongest effects on asthma (adjusted OR 2.46, 95% CI 1.19–5.08), chronic bronchitis (adjusted OR 1.45, 95% CI 1.08–1.96) and respiratory symptoms, such as wheezing (adjusted OR 1.30, 95% CI 0.90–1.89). The associations were weaker for exposure during early-life (adjusted ORs 1.38/1.27/1.15 respectively) and after 2 years of age (adjusted ORs 1.45/1.34/1.18) compared to prenatal exposure and the weakest or non-existent for current exposure (adjusted ORs 1.05/1.09/1.06). Upper respiratory infections were associated more strongly with early-life exposure (adjusted OR 1.25, 95% CI 1.09–1.42) than with prenatal (adjusted OR 0.74, 95% CI 0.54–1.01) or current exposure (adjusted OR1.05, 95% CI 0.92–1.20). The risk of allergies was also related to early life exposure to tobacco smoke (adjusted OR 1.26, 95% CI 1.13–1.42).

**Conclusion:**

Adverse effects of tobacco smoke on asthma, chronic bronchitis, and chronic respiratory symptoms are strongest when smoking takes place during pregnancy. The relations are weaker for exposure during early-life and after 2 years of age and weakest or non-existent for current exposure.

## Background

There is strong evidence that maternal smoking during pregnancy is harmful to fetal development. Tobacco smoke constituents pass placenta and reduce intrauterine fetal growth and increases the risk of preterm delivery [1]. There is also accumulating evidence that maternal smoking in pregnancy may influence the fetal development of respiratory system, suggested by findings of a relation between maternal smoking in pregnancy and lung function impairment in newborns [2-7]. Based on a recent review, there is strong evidence that exposure to environmental tobacco smoke (ETS) in childhood causes chronic respiratory symptoms, such as cough, phlegm, and wheezing, susceptibility to lower respiratory infections and to acute and recurrent otitis media, and that it has a causal role in childhood asthma [8]. Evidence of the effects of ETS exposure on allergies is inconsistent [8]. There is also evidence that maternal smoking in pregnancy increase the risk of asthma [8-11] and wheezing [11,12] in childhood. Only few studies have assessed the relative impact of prenatal and postnatal exposure to tobacco smoke on the child's later asthma or chronic respiratory symptoms [10,12], and to our knowledge no studies have elaborated respiratory infections and allergies in this context.

We assessed the independent and joint effects of prenatal and postnatal exposure to tobacco smoke on the risk of asthma and other respiratory problems at school age in a study of Russian school children in 9 cities. We also elaborated whether the effect of smoking in pregnancy on respiratory problems is mainly mediated through reduced fetal growth and duration of pregnancy.

## Methods

### Study design and population

We conducted a cross-sectional study, which was designed to assess the effects of air pollution on children's respiratory health. The study population was recruited through primary schools located in the vicinity of air pollution monitoring stations in 12 areas in 9 Russian cities in the Middle Urals and Upper Volga regions [13]. One school from each area was selected. The study population comprised 5951 2–5^th ^graders aged 8 to 12 years. The response rate in schools varied from 96% to 98%. The questionnaire, modified from previous European and North American questionnaires for the Russian conditions [14-16], inquired about the child's personal characteristics, health information, and socioeconomic factors. Local elementary school teachers were trained to instruct the parents about filling out the questionnaires, and parents and guardians were invited to meetings after the school day. After signing an informed consent, a parent completed the questionnaire.

### Health outcomes

The main health outcomes were asthma (ever, current), allergies (any, respiratory), chronic respiratory symptoms (wheezing, cough, phlegm), chronic bronchitis (doctor diagnosed ever, current) and upper respiratory infections (any, severe). In addition we constructed a composite variable called "asthma-like symptoms". These are defined in detail in Table 1.

**Table 1:** Prevalence of the respiratory health outcomes in the study population. The 95% confidence intervals are based on binomial distribution.

**Health outcome**	**Definition**	**Prevalence (95% CI)**
**Doctor diagnosed asthma**		
Ever	Ever told by doctor that the child has asthma	1.9 (1.5 – 2.3)
Current	Diagnosed by doctor with shortness of breath, wheeze, or use of asthma medication within past 12 months	1.5 (1.2 – 1.8)
**Asthma like symptoms**	Asthma symptoms or asthma medication use, awakening by asthma, wheezing upon exercise, or hospital care for wheezing within past 12 months	10.3 (9.5 – 11.1)
**Wheezing**		
Ever without cold	Wheezing heard from distance without a cold	3.1 (2.6 – 3.5)
Current	Wheezing heard from distance with or without a cold, shortness of breath with wheezing, awakening at night by wheezing, wheezing with exercise, or use of medication or hospitalisation within past 12 months for wheezing	13.4 (12.5 – 14.3)
**Cough**		
Any ever	Usual cough day or night	25.7 (24.6 – 26.9)
Any persistent	Cough ≥ 3 consecutive months within past 12 months	5.5 (4.9 – 6.1)
**Phlegm**		
Ever	Wet cough or phlegm produced without a cold	7.0 (6.3 – 7.7)
Persistent	Wet cough or phlegm ≥ 3 consecutive months within past 12 months	1.5 (1.2 – 1.9)
**Doctor diagnosed bronchitis ever**	Ever told by doctor that child has bronchitis	25.6 (24.5 – 26.8)
**Current bronchitis**	Doctor-diagnosed bronchitis within past 12 months	8.3 (7.6 – 9.0)
**Respiratory infections**		
Upper respiratory infection	Acute upper respiratory infection within past 12 months	76.9 (75.8 – 78.0)
Severe upper respiratory infection	Two or more acute upper respiratory infections within past 12 months	24.2 (23.1 – 25.3)
**Allergy**		
Any	Doctor-diagnosed allergy, reported hay fever, or pollenosis	33.2 (32.0 – 34.4)
Respiratory	Hay fever or doctor-diagnosed allergies to airborne substances (e.g., dust, animals, molds, pollens, air pollution, tobacco smoke)	8.0 (7.3 – 8.7)

### Exposure assessment

Exposure assessment was based on questionnaire information on maternal smoking during pregnancy (prenatal exposure), exposure to tobacco smoke during the first two years of life (early-life exposure), after the age of 2, and at the time of the survey (current exposure). Table 2 shows the definitions of the exposure parameters and the prevalences of exposure in the study population.

**Table 2:** Exposure to tobacco smoke in Russian school children (N = 5971).

**Exposure**	**Definition**	**Prevalence (95% CI)**
Fetal exposure: Mother smoked during pregnancy	Did this child's mother smoke while she was pregnant with this child? If yes: (A) Specify in figures number of cigarettes per week if she was an occasional smoker. (B) Specify in figures number of cigarettes per day if she smoked every day.	4.3 (3.8 – 4.9)
Early-life exposure: ETS when child was younger that 2 years	Between the times this child was born and he or she turned 2 years old, were there any smokers in regular contact with the child? Include regular visitors, for example, grandparents or baby-sitters. If yes: Did this child's mother (or stepmother or other female taking care of the child) smoke during this period?	46.5 (45.2 – 47.7)
ETS when child after 2 years of age	Between the times the child turned 2 years old and he or she started school, were there any smokers in regular contact with the child? Include regular visitors, for example, grandparents or baby-sitters. If yes: Did this child's mother (or stepmother or other female taking care of the child) smoke during this period?	51.1 (49.8 – 52.4)
Current smokers in the household	Does anyone daily smoke cigarettes, papirosy (Russian non-filter cigarette), cigars, or pipes in this child's home? If yes: (A) On average, how many cigarettes or papirosy, in total, are smoked in the home each day when the child is at home? (B) On average, how many cigars are smoked in the home each day while the child is at home? (C) On average, how many pipes are smoked in the home each day while the child is at home?	46.1 (44.9 – 47.4)
Prenatal exposure only		0.2 (0.1 – 0.3)
Postnatal exposure only		59.8 (58.6 – 61.1)
Both prenatal and postnatal exposure		4.1 (3.6 – 4.7)
Any tobacco smoke exposure	Any of the above.	64.5 (63.3 – 65.7)

### Statistical methods

We estimated the prevalences (%) of the respiratory outcomes and exposure with 95 % confidence intervals based on the binomial distribution. Odds ratio was the measure of effect. We used logistic regression analysis to estimate adjusted odds ratios for the relations between exposure to tobacco smoke, and pregnancy and respiratory outcomes. The basic adjustment was made using the following core covariates: study area, age, gender, mother's education (low, medium, high), and parental asthma. We fitted also income, furry pets and sharing a bedroom as covariates, but excluded them if they changed the studied estimates less than 10%. Additional adjustment was made for low birth weight and preterm delivery when studying the relations between maternal smoking and respiratory outcomes. Maternal smoking was included as an additional covariate when studying the relations of low birth weight and preterm delivery to respiratory outcomes.

## Results

### Study population

Table 3 describes the characteristic of the study population according to exposure to tobacco smoke. The age and sex distributions were similar among the exposed and the reference group, but the exposed were more likely to have mother with low education, to be born prematurely, have lower birth weight, and to be exposed to furry or feathery pets.

**Table 3:** Characteristics of the study population by exposure to tobacco products, either through maternal smoking in pregnancy or environmental tobacco smoke in lifetime.

**Characteristic**	**Exposed, N (%)**	**Reference, N (%)**	**Total, N(%)**
Total	3790 (64.5)	2084 (35.5)	5874
Age			
8	561 (14.8)	317 (15.2)	878 (14.9)
9	1268 (33.5)	675 (32.4)	1943 (33.1)
10	1216 (32.1)	654 (31.4)	1870 (31.8)
11	647 (17.1)	396 (19.0)	1043 (17.8)
12	98 (2.6)	42 (2.0)	140 (2.4)
Gender, boy	1918 (50.6)	1053 (50.5)	2971 (50.6)
Mother's education			
Higher	636 (16.9)	492 (23.7)	1128 (19.3)
Incomplete higher or college	2070 (55.0)	1102 (53.2)	3172 (54.4)
Secondary	1056 (28.1)	479 (23.1)	1535 (26.3)
Parental asthma	66 (1.8)	41 (2.0)	107 (1.8)
Low birth weight ( ≤ 2500 g)	248 (6.9)	109 (5.5)	357 (6.4)
Premature birth ( < 37 weeks)	390 (10.5)	172 (8.3)	562 (9.7)
Income below average	2404 (63.9)	1259 (60.8)	3663 (62.8)
Others sleeping in the same room with child	2760 (73.5)	1490 (71.9)	4250 (72.9)
Furry or feathery pets	2421 (65.6)	1247 (61.4)	3668 (64.1)

### Maternal smoking in pregnancy and the risk of adverse pregnancy outcomes

Only 4.3% of the mothers reported having smoked during pregnancy. Maternal smoking was a determinant of both preterm delivery and low birth weight. The risk of low birth weight was higher among newborns of smoking women compared to those of non-smokers with an adjusted odds ratio of 1.34 (95% CI 0.82–2.18). The corresponding effect estimate for preterm delivery was 1.85 (95% CI 1.30–2.68).

### Fetal growth, preterm delivery and the risk of respiratory outcomes

Both preterm delivery and low birth weight increased the risk of asthma and asthma-like symptoms, chronic respiratory symptoms, and doctor-diagnosed bronchitis, as shown in Table 4. The risk of severe upper respiratory infections was related to preterm delivery (adjusted OR 1.21, 95% CI 0.99–1.48), but not to low birth weight (adjusted OR 1.01, 95% CI 0.79–1.30). The risk of allergies was not related to preterm delivery, but interestingly the risk of any allergy was significantly lower among children with low birth weight (adjusted OR 0.78, 95% CI 0.61–0.99).

**Table 4:** Crude and adjusted odds ratios for respiratory health outcomes according to low birth weight and preterm delivery.

**Health outcome**	**Preterm delivery ( < 37 weeks)**		**Low birth weight ( < 2500 g)**	
	
	**Crude OR (95% CI)**	**Adjusted ^1 ^OR (95% CI)**	**Crude OR (95% CI)**	**Adjusted ^1 ^OR (95% CI)**
**Doctor diagnosed asthma**				
Ever	1.80 (1.06 – 3.04)	1.96 (1.14 – 3.35)	1.73 (0.92 – 3.27)	1.88 (0.98 – 3.59)
Current	1.83 (1.00 – 3.34)	1.95 (1.06 – 3.62)	1.93 (0.95 – 3.89)	2.05 (0.99 – 4.22)
**Asthma like symptoms**	1.47 (1.13 – 1.90)	1.48 (1.14 – 1.93)	1.38 (1.00 – 1.90)	1.43 (1.03 – 1.99)
**Wheezing**				
Ever without cold	1.54 (0.99 – 2.40)	1.62 (1.04 – 2.54)	1.67 (1.00 – 2.79)	1.76 (1.04 – 2.96)
Current	1.28 (1.00 – 1.63)	1.30 (1.01 – 1.66)	1.19 (0.88 – 1.62)	1.24 (0.91 – 1.69)
**Cough**				
Ever	1.38 (1.14 – 1.67)	1.38 (1.13 – 1.68)	1.22 (0.96 – 1.56)	1.27 (0.99 – 1.62)
Persistent	1.78 (1.28 – 2.46)	1.86 (1.34 – 2.59)	1.66 (1.12 – 2.48)	1.72 (1.15 – 2.58)
**Phlegm**				
Ever	1.21 (0.87 – 1.68)	1.23 (0.88 – 1.71)	1.15 (0.76 – 1.73)	1.18 (0.78 – 1.78)
Persistent	2.52 (1.47 – 4.34)	2.73 (1.57 – 4.76)	2.13 (1.09 – 4.16)	2.26 (1.14 – 4.47)
**Doctor diagnosed bronchitis ever**	1.50 (1.24 – 1.81)	1.55 (1.28 – 1.88)	1.53 (1.22 – 1.93)	1.63 (1.29 – 2.06)
**Current bronchitis**	1.49 (1.12 – 1.97)	1.52 (1.14 – 2.02)	1.28 (0.89 – 1.84)	1.32 (0.91 – 1.92)
**Respiratory infections**				
Upper respiratory infection	1.10 (0.89 – 1.36)	1.11 (0.89 – 1.38)	1.06 (0.81 – 1.38)	1.06 (0.81 – 1.39)
Severe upper respiratory infection	1.22 (1.00 – 1.48)	1.21 (0.99 – 1.48)	1.01 (0.79 – 1.30)	1.01 (0.79 – 1.30)
**Allergy**				
Any	1.01 (0.84 – 1.21)	1.04 (0.86 – 1.26)	0.78 (0.62 – 0.99)	0.78 (0.61 – 0.99)
Respiratory	1.13 (0.83 – 1.55)	1.14 (0.83 – 1.57)	0.93 (0.62 – 1.40)	0.94 (0.62 – 1.41)

^1 ^– Odds ratios adjusted for study area, age, gender, mother's education, parental asthma, maternal smoking during pregnancy.

### Prenatal and postnatal exposure and the risk of respiratory outcomes

Table 5 shows the relations between prenatal, early-life, after 2 years of age, and current exposures and the risk of various health outcomes. The effect estimates are displayed adjusted for the core covariates and further for preterm delivery and low birth weight.

**Table 5:** Adjusted odds ratios for respiratory health outcomes according prenatal, early-life and current exposure.

**Health outcome**	Prenatal exposure		Early-life exposure		Exposure after age of 2		Current exposure	
	**Adjusted OR ^1 ^(95% CI)**	**Adjusted OR ^2 ^(95% CI)**	**Adjusted OR ^1 ^(95% CI)**	**Adjusted OR^2 ^(95% CI)**	**Adjusted OR ^1 ^(95% CI)**	**Adjusted OR ^2 ^(95% CI)**	**Adjusted OR ^1 ^(95% CI)**	**Adjusted OR ^2 ^(95% CI)**
**Doctor diagnosed asthma**								
Ever	2.40 (1.17 – 4.93)	2.46 (1.19 – 5.08)	1.39 (0.94 – 2.06)	1.38 (0.93 – 2.06)	1.45 (0.97 – 2.17)	1.45 (0.96 – 2.18)	1.10 (0.74 – 1.63)	1.05 (0.70 – 1.58)
Current	2.55 (1.13 – 5.78)	2.64 (1.16 – 6.03)	1.35 (0.87 – 2.12)	1.36 (0.86 – 2.15)	1.44 (0.91 – 2.27)	1.44 (0.90 – 2.32)	1.19 (0.75 – 1.87)	1.09 (0.68 – 1.74)
**Asthma like symptoms**	1.48 (1.01 – 2.15)	1.39 (0.93 – 2.08)	1.28 (1.07 – 1.52)	1.26 (1.05 – 1.51)	1.23 (1.03 – 1.47)	1.22 (1.02 – 1.47)	1.09 (0.91 – 1.30)	1.09 (0.90 – 1.30)
**Wheezing**								
Ever without cold	0.74 (0.30 – 1.83)	0.59 (0.21 – 1.63)	1.24 (0.91 – 1.69)	1.24 (0.91 – 1.70)	1.40 (1.02 – 1.92)	1.42 (1.03 – 1.96)	1.00 (0.73 – 1.38)	1.00 (0.72 – 1.37)
Current	1.37 (0.97 – 1.94)	1.30 (0.90 – 1.89)	1.18 (1.01 – 1.38)	1.15 (0.98 – 1.36)	1.20 (1.03 – 1.41)	1.18 (1.00 – 1.39)	1.09 (0.93 – 1.28)	1.06 (0.90 – 1.25)
**Cough**								
Ever	1.51 (1.13 – 2.02)	1.54 (1.14 – 2.08)	1.35 (1.2 – 1.53)	1.34 (1.18 – 1.52)	1.49 (1.31 – 1.68)	1.46 (1.28 – 1.66)	1.33 (1.18 – 1.51)	1.33 (1.17 – 1.51)
Persistent	1.11 (0.63 – 1.95)	0.98 (0.53 – 1.8)	1.27 (1.00 – 1.6)	1.20 (0.94 – 1.53)	1.27 (1 – 1.62)	1.23 (0.96 – 1.58)	1.24 (0.98 – 1.58)	1.19 (0.93 – 1.52)
**Phlegm**								
Ever	0.98 (0.58 – 1.65)	0.98 (0.57 – 1.69)	1.15 (0.93 – 1.41)	1.15 (0.92 – 1.42)	1.39 (1.13 – 1.72)	1.38 (1.11 – 1.71)	1.31 (1.06 – 1.62)	1.37 (1.1 – 1.7)
Persistent	0.56 (0.14 – 2.34)	0.56 (0.13 – 2.35)	1.33 (0.85 – 2.07)	1.33 (0.84 – 2.11)	1.68 (1.05 – 2.68)	1.74 (1.07 – 2.84)	0.82 (0.52 – 1.31)	0.84 (0.52 – 1.35)
**Doctor-diag bronchitis ever**	1.41 (1.06 – 1.88)	1.45 (1.08 – 1.96)	1.28 (1.14 – 1.45)	1.27 (1.12 – 1.44)	1.33 (1.18 – 1.51)	1.34 (1.18 – 1.52)	1.12 (0.99 – 1.26)	1.09 (0.96 – 1.24)
**Current bronchitis**	1.71 (1.14 – 2.56)	1.63 (1.06 – 2.49)	1.06 (0.87 – 1.28)	1.04 (0.85 – 1.27)	1.11 (0.92 – 1.34)	1.10 (0.90 – 1.34)	1.01 (0.83 – 1.22)	0.96 (0.79 – 1.18)
**Respiratory infections**								
Upper respiratory infection	0.77 (0.57 – 1.03)	0.74 (0.54 – 1.01)	1.25 (1.10 – 1.41)	1.25 (1.09 – 1.42)	1.28 (1.13 – 1.45)	1.29 (1.12 – 1.47)	1.04 (0.92 – 1.18)	1.05 (0.92 – 1.20)
Severe upper respiratory infection	1.21 (0.90 – 1.63)	1.14 (0.83 – 1.56)	1.24 (1.10 – 1.40)	1.23 (1.09 – 1.40)	1.18 (1.04 – 1.33)	1.18 (1.04 – 1.34)	1.04 (0.92 – 1.17)	1.05 (0.93 – 1.19)
**Allergy**								
Any	0.98 (0.74 – 1.31)	1.00 (0.74 – 1.35)	1.30 (1.16 – 1.46)	1.26 (1.13 – 1.42)	1.20 (1.07 – 1.35)	1.18 (1.05 – 1.33)	1.12 (1 – 1.25)	1.11 (0.99 – 1.25)
Respiratory	1.14 (0.70 – 1.85)	1.13 (0.68 – 1.87)	1.24 (1.02 – 1.51)	1.20 (0.99 – 1.47)	1.10 (0.90 – 1.34)	1.09 (0.89 – 1.33)	1.05 (0.86 – 1.28)	1.05 (0.86 – 1.29)

1 Logistic regression analysis: adjusted for the core covariates including study area, age, gender, mother's education, and parental asthma.

2 Logistic regression analysis: adjusted for the core covariates, preterm delivery and low birth weight.

Maternal smoking during pregnancy had a strong consistent effect on the risk of doctor-diagnosed asthma (ever: adjusted OR 2.46, 95% CI 1.19–5.08), asthma-like symptoms (adjusted OR 1.39 95% CI 0.93–2.08), current wheezing (adjusted OR 1.30, 95% CI 0.90–1.89), cough (adjusted OR 1.54, 95% CI 1.14–2.08) and doctor diagnosed bronchitis (ever: adjusted OR 1.45, 95% CI 1.08–1.96). The occurrence of phlegm, upper respiratory infections and allergy were not related to maternal smoking in pregnancy.

The effect estimates of early-life exposure for asthma and asthma like symptoms were lower than corresponding estimates for maternal smoking in pregnancy, varying from 1.04 to 1.38. The effect estimates of current asthma (adjusted OR 1.35, 95% CI 0.86–2.15), asthma ever (adjusted OR 1.38, 95% CI 0.93–2.06) and current wheezing (adjusted OR 1.15, 95% CI 1.01–1.38) were elevated, although not reaching statistical significance. That of asthma-like symptoms (adjusted OR 1.26, 95% CI 1.05–1.51) was statistically significant. Adjustment for preterm delivery and low birth weight did not alter the effect estimates. The risk of cough (ever: adjusted OR 1.34, 95% CI 1.18–1.52), doctor diagnosed bronchitis (adjusted OR 1.27, 95% CI 1.12–1.44), upper respiratory infections (adjusted OR 1.25, 95% CI 1.09–1.42) as well as allergies (any: adjusted OR 1.26, 95% CI 1.13–1.42; respiratory: adjusted OR 1.20, 95% CI 0.99–1.47) were also related to early-life exposure to ETS. The effect estimates for exposure after 2 years of age were similar to those of early-life exposure.

The risk of asthma, asthma like symptoms, wheezing, and upper respiratory infections were not related to current exposure to ETS, but the effect estimates for cough (any adjusted OR 1.33, 95% CI 1.17–1.51) and doctor diagnosed bronchitis (adjusted OR 1.09, 95% CI 0.96–1.24) as well as any allergy (adjusted OR 1.11, 95% CI 0.99–1.25) were elevated.

Table 6 compares the effects of prenatal exposure only, postnatal exposure only and both prenatal and postnatal exposure. In general the effect of experiencing both prenatal and postnatal exposure was stronger than the effect of postnatal exposure only. There were only 10 individuals who were exposed only during pregnancy and therefore the estimates are either not available or with wide confidence intervals. Therefore the comparison of independent effects of prenatal and postnatal exposures was possible for only few outcomes. The effects of prenatal exposure appeared to be stronger for asthma like symptoms and bronchitis.

**Table 6:** Adjusted odds ratios for respiratory health outcomes according prenatal exposure only (n = 10), postnatal exposure only (n = 3491) and both prenatal and postnatal exposure (n = 242).

**Health outcome**	**Prenatal exposure only Adjusted OR ^1^(95% CI)**	**Postnatal exposure only Adjusted OR ^1 ^(95% CI)**	**Both prenatal and postnatal exposure Adjusted OR ^1 ^(95% CI)**
**Doctor diagnosed asthma**			
Ever	NA ^2^	1.20 (0.77 – 1.87)	2.96 (1.35 – 6.51)
Current	NA ^2^	1.32 (0.79 – 2.23)	3.48 (1.41 – 8.56)
**Asthma like symptoms**	1.53 (0.18 – 12.77)	1.26 (1.03 – 1.54)	1.64 (1.07 – 2.53)
**Wheezing**			
Ever without cold	NA ^2^	1.32 (0.94 – 1.86)	0.75 (0.27 – 2.13)
Current	1.07 (0.13 – 8.91)	1.21 (1.02 – 1.45)	1.51 (1.01 – 2.24)
**Cough**			
Ever	1.39 (0.28 – 7.00)	1.42 (1.23 – 1.63)	1.96 (1.42 – 2.70)
Persistent	NA ^2^	1.30 (0.99 – 1.70)	1.19 (0.63 – 2.25)
**Phlegm**			
Ever	NA ^2^	1.37 (1.08 – 1.74)	1.25 (0.70 – 2.21)
Persistent	NA ^2^	1.53 (0.90 – 2.59)	0.77 (0.17 – 3.39)
**Doctor diagnosed bronchitis ever**	3.50 (0.84 – 14.52)	1.34 (1.17 – 1.54)	1.75 (1.27 – 2.40)
**Current bronchitis**	1.64 (0.18 – 14.58)	1.16 (0.93 – 1.43)	1.81 (1.15 – 2.87)
**Respiratory infections**			
Upper respiratory infection	0.47 (0.11 – 2.06)	1.27 (1.11 – 1.47)	0.89 (0.64 – 1.24)
Severe upper respiratory infection	0.51 (0.06 – 4.21)	1.20 (1.05 – 1.37)	1.33 (0.95 – 1.85)
**Allergy**			
Any	2.31 (0.55 – 9.66)	1.26 (1.12 – 1.43)	1.14 (0.83 – 1.57)
Respiratory	NA ^2^	1.14 (0.92 – 1.42)	1.32 (0.78 – 2.22)

^1 ^Logistic regression analysis: adjusted for the core covariates, preterm delivery and low birth weight.

^2 ^Estimate not available due to small number of exposed cases.

## Discussion

Our population-based epidemiologic study in nine Russian cities shows the harmful effects of fetal and early-life exposure to tobacco smoke products. Prenatal exposure due to maternal smoking had the strongest effects on asthma, chronic bronchitis and respiratory symptoms. The associations were weaker for exposure during early-life and after 2 years of age and weakest or non-existent for current exposure. Upper respiratory infections were associated more strongly with early-life exposure than with prenatal exposure. The risk of allergies was also weakly related to both prenatal and postnatal exposure to tobacco smoke.

Although maternal smoking was, as expected, a strong determinant of preterm delivery and low birth weight, and these adverse pregnancy outcomes were strong predictors of asthma and other respiratory problems, adjustment for preterm delivery and low birth weight had little influence on the associations between prenatal tobacco smoke exposure and respiratory outcomes.

### Validity of results

We achieved a very high response rate due to strong support by the parents and teachers and this practically eliminates selection bias related to participation. Information on exposure to tobacco smoke was collected retrospectively and there is a possibility for both random and systematic errors. Maternal smoking during pregnancy was reported to be much lower (4%) compared to maternal smoking after the delivery (12 %). This could reflect problems in recall or cultural behavior to quit smoking during pregnancy. The latter alternative is supported by our similar findings in another Russian female population in Karelia, North-West Russia (J Jaakkola, unpublished observation), where smoking during pregnancy was 5% whereas the smoking was 25% when the child was at school. Observed associations between maternal smoking and low birth weight, which are consistent with previously published meta-analyses [1] and research reports [9], support the validity of information on prenatal exposure indirectly. Given the nature of the study design we can not fully exclude the possibility of non-comparable exposure information from parents of ill and healthy children. We were able to adjust the effect estimates for several potential confounders.

### Synthesis with previous knowledge

Only few previous studies have elaborated the relative contributions of prenatal and postnatal exposures to ETS on asthma [10,11], and chronic respiratory symptoms [11,12], and to our knowledge no studies have focused on respiratory infections or allergies.

Our findings on the stronger effect of prenatal exposure on asthma and wheezing compared with postnatal exposure are consistent with the results of a cross-sectional study of 11,500 8 to 11 years old children in 24 US and Canadian communities [11]. A recent cross-sectional study of 5 762 Californian school children [10] also provided evidence of the relative importance of prenatal exposure in development of asthma with a retrospective recording of in utero and previous postnatal and current exposure. In utero exposure to maternal smoking without subsequent postnatal exposure to ETS was related to the presence of asthma in 4^th^, 7^th^, and 10^th ^grade children with an adjusted odds ratio of 1.8 (95% CI 1.1 – 2.9). In contrast, current or previous postnatal exposure to ETS was not associated with asthma risk, but the risk of lifetime wheezing was increased with an odds ratio of 1.3 (95% CI 1.1 – 1.5). Lux and colleagues showed in a longitudinal study of 8561 English children that maternal smoking during pregnancy causes wheezing during the first 30 months of life independently from postnatal exposure [11]. A large population-based cohort study of 55,000 Finnish children estimated a 35% increased risk of asthma by the age of 7 years related to maternal smoking of over 10 cigarettes per day during pregnancy [9]. Similarly to the present study, maternal smoking was a strong determinant of both preterm delivery and low birth weight, and these adverse pregnancy outcomes were strong predictors of asthma, but practically none of the effect of maternal smoking on asthma was mediated via the pregnancy outcomes.

In the present study prenatal exposure had also a stronger effect on both lifetime and current chronic bronchitis compared with early life exposure and there was no association between current exposure and chronic bronchitis. To our knowledge this has not been reported before. However, the occurrence of upper respiratory infections during the previous year was related to early life exposure to ETS, but not to prenatal or current exposure.

Our results strengthen the evidence that also postnatal exposure to tobacco smoke increases the risk of asthma in childhood. The effect estimates for early-life exposure and exposure after the age of two were consistently elevated suggesting a 30% increase in risk, although there was no association between current exposure and asthma. The latter could be explained by avoidance of smoking in the presence of the child after the diagnosis of asthma. Unfortunately the number of children with prenatal exposure only was too small to get good estimates of the independent effect of prenatal exposure only. However the effect of both prenatal and postnatal exposure on asthma was much stronger (adjusted OR 3.48) than the effect of postnatal exposure only (adjusted OR 1.32), suggesting a synergistic effect of prenatal and postnatal exposures. There is evidence that maternal smoking in pregnancy reduces the fetal development of lung function [2-7], which may play a role in the susceptibility to the effects of exposure to environmental tobacco smoke after delivery. Maternal smoking in pregnancy may also have other effects increasing the susceptibility, including effects on development and maturation of the pulmonary immune system [17] leading to an increased bronchial reactivity in early childhood as shown by Young and colleagues [7].

Early life exposure to ETS was a determinant of both any and respiratory allergy at school age. There was a weak non-significant association between prenatal exposure and respiratory allergy and no association with any allergy. There was also a weak positive association between current exposure and risk of any allergy. In their systematic review based on 36 relevant articles in 1998, Strachan and Cook [18] concluded that parental smoking, either before or immediately after birth, is unlikely to increase the risk of allergic sensitisation in children. In a Norwegian study, a negative association between prenatal smoking and childhood atopy was found, but rather than proposing a causal relation the investigators suggest that selective avoidance of smoking during pregnancy is an alternative explanation [19].

### Concluding remarks

This large epidemiologic study in Russian children confirms that smoking during pregnancy and in the presence of children is harmful for respiratory health increasing the risk of asthma, chronic bronchitis, and respiratory infections and possibly allergies. The results strengthen the evidence that the adverse effects of tobacco smoke on asthma, chronic bronchitis, and chronic respiratory symptoms are strongest when smoking takes place during pregnancy and stronger during early life compared with exposure in school age. Although maternal smoking reduces duration of gestation and fetal growth and these pregnancy outcomes predict respiratory illness, little of the effect of maternal smoking in pregnancy on respiratory health is mediated via preterm delivery and low birth weight. In conclusion, smoking in pregnancy and in the presence of children is among the most serious preventable hazards to children's health.

## Authors' contributions

JJ conceived the hypothesis, participated in the planning of the study and statistical analyses, and wrote the paper. AK conducted the statistical analyses and contributed to the interpretation of the results and writing of the paper. BK participated in the planning of the study and supervision of the data collection, and contributed to the writing of the paper. SK and LP participated in the planning of the study and supervision of the data collection. JS designed and led the study and contributed to the interpretation of the results and writing of the paper. All authors read and approved the final manuscript.
